# Hints to the Editors

**Published:** 1816-04

**Authors:** 


*For the London Medical and Physical Journal.*

*Hints to the Editors;* by A. Z.

YOU have exemplified, in some criticisms in your last Number on cruel and useless experiments, that "authors, before they write, should read." It would, indeed, save them much unnecessary trouble, and much valuable luable time to their readers. Too many are so fond of appearing in print, or so desirous of converting a book into an advertisement, that the press is loaded with knowledge familiar to every one. Even your valuable publication is sometimes taxed with such unnecessary information: for who, in these enlightened days, requires to be informed that large and repeated venæsection is a principal remedy in pleurisies,—or that vaccination is not always a security against the small-pox? Yet, I believe, few well-informed practitioners doubt, if the last was universally practised at an early age, that the small-pox would, in a few years, be unknown. I hope, therefore, that gentlemen will be more sparing of their communications, unless they have some new observations to offer, or some practice to reform. Space will then remain for the insertion of valuable information from various medical and philosophical publications, both at home and abroad,—publications which cannot be generally procured in this country. I am an old practitioner, yet I am still eager for information; but cannot want to be told what almost every medical author has written. Medical knowledge is progressive, and therefore time should not be wasted on subjects well known to every reader.

*Feb.* 18, 1816.

---

We are extremely obliged by the judicious remarks of our venerable correspondent, and the more so, as they contain hints we should feel a backwardness in offering to gentlemen to whom we are under obligations for so many former valuable practical lessons. We trust A. Z. does not mean to accuse us of want of diligence in collecting all the information in our power from books, from reports, and even from conversations. Whenever he sees occasion to remind us of deficiency in any of these points, we shall thankfully avail ourselves of his instructions.

Meanwhile, from so old a practitioner, and from one so eager for information, we cannot help expressing a wish for some useful communication, not only for the amusement of his Nestorean brethren, but for the advantage of our younger correspondents and readers.—Edit.

